# Factors Affecting Sport-Related Concussion Non-Disclosure in Women’s Rugby—A Multi-Country Qualitative Analysis

**DOI:** 10.3390/jfmk9040277

**Published:** 2024-12-18

**Authors:** Lisa Ryan, Ed Daly, Katherine Hunzinger

**Affiliations:** 1 Department of Sport, Exercise and Nutrition, School of Science and Computing, Atlantic Technological University, Dublin Rd., H91 T8NW Galway, Ireland; ed.daly@atu.ie; 2 Department of Exercise Science, Thomas Jefferson University, Philadelphia, PA 19144, USA; katherine.hunzinger@jefferson.edu; 3 Jefferson Center for Injury Research & Prevention, Thomas Jefferson University, Philadelphia, PA 19144, USA

**Keywords:** female athlete, injury reporting, collision sport, mild traumatic brain injury, rugby code

## Abstract

**Background and Objectives:** Collision-sport athletes, such as rugby players, are at risk of sport-related concussion (SRC). Women are known to be at higher risk of SRC and may experience more severe and chronic symptomology than men. Knowledge of the factors that affect a player’s disclosure of their concussive symptoms could help to inform strategies to improve compliance with reporting and management of head injuries. The aim of this study was to investigate the factors that may impact women rugby players’ disclosure of a concussion. **Methods**: Twenty-eight adult (≥18 years of age) elite and semi-elite women rugby players from the UK and Ireland (*n* = 17) and the United States (*n* = 11) were interviewed on their playing background and SRC experience in women’s rugby via one-on-one interviews (UK and Ireland) or an online questionnaire (US). **Results**: SRC data were analysed inductively using a thematic analysis approach to determine the potential reasons for SRC non-disclosure in women’s rugby. Four main themes were identified which may influence a player’s SRC non-disclosure: 1. women rugby players are self-managing SRC; 2. work-related concerns impact on SRC disclosure; 3. players and support staff lack knowledge on SRC management; and 4. poor internal and external communication affect the support players receive when injured. **Conclusions:** The findings were consistent across players from different countries. This research highlighted several factors that may impact on women rugby players’ disclosure of SRC regardless of country of origin (UK, Ireland, or US) and access to concussion care. Coaches and management teams should be aware of these reasons, which may enhance how they discuss and manage concussion. There is a clear need for further education on concussion for players and support staff and for strategies to create environments where women can openly discuss their concussion concerns.

## 1. Introduction

Women’s rugby is a rapidly growing sport; there has been a 28% increase in registered players globally since 2017, and women now comprise over 25% of the total rugby-playing population [1]. While it is encouraging to see the continued increase in women playing rugby, as rugby is a collision sport, with increased participation comes an increased risk of injury, including sport-related concussion (SRC). Concussion in sport is a very complex, heterogenous injury that may cause impairment across multiple domains of neurological function [2,3,4,5]. There is currently no universally agreed definition of concussion. The definitions vary due to differences across organisations, medical disciplines and research areas; a lack of consensus in the literature; and the rapid development of research in this area [6,7,8,9,10,11]. The Concussion in Sport Group (CISG) most recently defined a SRC as ‘a traumatic brain injury (TBI) caused by a direct blow to the head, neck or body resulting in an impulsive force being transmitted to the brain that occurs in sports and exercise-related activities’ [7]. It has been suggested that women may have a lower acceleration threshold than men to sustain a SRC [11]; however, there is no defined threshold for SRCs given the diversity of factors that may contribute to the risk of a SRC.

SRCs are more commonly recognised by a number of signs and symptoms which can vary in presentation and severity between players. Symptoms may present as cognitive impairments [12], sleeping irregularities [13], mood deficits [14], post-traumatic migraine [15], cervicogenic injuries [16], vestibular and ocular deficits [17], or autonomic nervous system disturbances [18]. SRC symptoms may present immediately or develop over time and typically resolve within several days, but they may be prolonged, leading to chronic symptomology [7]. The inconsistent presentation of SRC presents a considerable difficulty in its diagnosis, not least because there are still no objective measures for concussion identification. As a result of the subjective nature of SRC, medical teams often rely on players’ reporting of symptoms. Concussion disclosure and the factors that may impact this disclosure are therefore of considerable interest.

Research has previously investigated some of the factors that may impact concussion reporting, such as concussion education/knowledge [19,20], motivation, intentions [21,22,23] and athlete identity [24,25]. To date, a considerable amount of the research has been quantitative in nature, without hearing the ‘athlete’s voice’. While quantitative data provide information on the physical effects of concussion, these measures do not enable us to gain an insight into the personal and emotional impact of concussive injury on athletes, which is essential to develop effective interventions. Several studies in this field have also overly focused on concussion knowledge, when evidence suggests that knowledge alone will not necessarily change concussion disclosure behaviours [26,27]. Research has also predominantly been conducted in men’s sports, with relatively few mixed-gender or women’s-only studies performed to date [28]. This is concerning given that the evidence to date suggests that women may be at greater risk of sustaining a SRC, take longer than men to recover from SRC and may be more prone to ongoing chronic symptoms [29,30]. The mechanisms and the rationale behind these differences are difficult to determine; however, Covassin et al. [30] previously summarised the known gender-differences in physiology and symptomology. Another emerging area to consider is the association between menstrual health and SRCs, with preliminary findings by Soligon et al. [31] suggesting a potential link between SRC history and menstrual irregularities.

Players who do not disclose a SRC and continue to play are at an increased risk of sustaining a second head injury which may lead to significant ongoing health implications [32]. In addition, research has also highlighted that those who continue to play after sustaining a concussion take longer to recover compared to those immediately removed from play [33]. Consequently, the disclosure of SRC has important ramifications for player welfare, SRC recovery and return-to-play (RTP) timelines. Understanding the rationale behind why women rugby players may not disclose a SRC is important for putting mechanisms in place to support enhanced reporting. Additionally, given the global nature of rugby, it is also important to understand if there are cross-cultural/cross-country differences in experience of concussion reporting. Therefore, the aim of this research was to investigate the ‘lived experience’ of women rugby players by using qualitative methods, including one-on-one interviews (United Kingdom (UK) and Ireland) and open-ended survey questions (United States (US)), to determine the reasons why women’s rugby union players choose not to disclose potential SRCs.

## 2. Methodology

### 2.1. Study Background

This study applied a reflexive thematic approach (RTA) integrated within a critical realist framework [34,35]. An RTA ensures that the researcher remains aware of their own influence on the analysis process. The ontological position of ‘critical realist’ is taken, where it is acknowledged that although a reality exists independently of the observer, we cannot know that reality with certainty, and therefore it balances a commitment to objective reality with a recognition of the limitations and socially embedded nature of our understanding [36]. The critical realist framework allowed for in-depth exploration to identify not just the experiences and events that were described by participants but also to try to identify the causal mechanisms behind these events to enhance our understanding of how different factors may influence how concussions are perceived, managed or treated. Semi-structured interviews were utilised to enable participants to reflect on their personal experiences with SRC [37,38]. This approach also enabled the researcher to explore responses with further probing questions where appropriate. The interview questions were designed to obtain responses on participants’ experience in women’s rugby, their individual playing background, and to establish their thoughts and opinions on SRC within women’s rugby and their personal experience. Participants were sought from the US, UK and Ireland to obtain cross-cultural perspectives as part of an ongoing collaboration on head injury in rugby.

### 2.2. Study Participants

UK and Ireland

Participants for the UK and Ireland part of the study were initially selected through purposive sampling via contacts of the lead researcher (LR), and further participants were accessed using snowball sampling. Three purposive sampling criteria were devised [39]. These were as follows: (1) participants had to be women (2) ≥18 years of age (3) playing elite or semi-elite rugby union in the UK and Ireland.

US

American participants were a subsample of a larger cohort of men and women American community rugby players (*n* = 1037) older than 18 years old with at least 1 year of tackle rugby (i.e., full-contact) playing history [40]. Participants from this cohort who were women and included responses in the open-ended text box for reason of concussion non-disclosure were included in this analysis.

### 2.3. Ethics and Procedures

UK and Ireland

For the 1-on-1 interviews (UK and Ireland), ethical approval, according to the Declaration of Helsinki, was granted to this study via the university ethics committee (RSC_GMIT). Invitations to participate were issued via email (by LR) alongside a participant information sheet and consent form. Participants were given the opportunity to discuss the aims of the study and how the research would be conducted. A variety of options for conducting the interviews were offered to each participant: face-to-face, online (via Teams, Zoom or Skype) or via telephone. Upon receiving notification of the preference, the lead researcher (LR) set up the interview date and time. Each participant provided informed consent prior to their interview. All interviews took place online via Microsoft Teams (Redmond, WA, USA) and were audio-recorded. Interviews were transcribed verbatim by the lead researcher (LR). Participants were asked if they would like to review their transcript. Ten out of the seventeen participants received their interview transcript by email within ten days of the interview to check for accuracy. There were no changes made to any transcripts.

US

American-based participants completed an online survey (Qualtrics, Provo, UT, USA) to ascertain intentionally unreported concussion history from rugby [40]. Participants were allowed the opportunity to provide follow-up information pertaining to this unreported injury via an open-ended text box. All participants provided informed consent prior to survey participation, as approved by the university’s Institutional Review Board.

### 2.4. Researcher Description

Reflexive thematic analysis recognises the active role of the researcher in the identification and interpretation of themes [34]. The researcher has a role in the creation of meaning and as such is not a passive observer but actively constructing themes based on their personal interpretation. The lead researcher (LR) of this study identifies as a woman and has been involved in higher education teaching in the UK, Ireland and/or Australia for 20 years. The lead researcher has been working with (men’s and women’s) rugby union teams for over 20 years and is the co-founder of the Irish Concussion Research Centre and a vocal advocate for women in sport. At the time of analysis, the lead researcher was actively working in women’s rugby as part of a larger coaching group. The lead researcher is an experienced qualitative researcher who has previously conducted similar interviews and analyses with male and female rugby players. The researcher’s background was reflected upon before data collection through the process of bracketing to mitigate any potentially negative effects of preconceptions on the research process and to increase the rigour of the process [41].

### 2.5. Data Analysis and Methodological Integrity

Data were thematically analysed utilising the approach developed by Braun and Clarke [34,42,43]. This six-phase guide to thematic analysis began with familiarisation with the data—transcribing the data from the recordings, rereading the interview transcripts and noting any initial ideas regarding the data as a whole [42]. Phase two involved generating initial codes from reviewing the data, highlighting meaningful text from the transcripts, collating them into a new data set relevant to the research topic and coding them based on the interesting features in the data. The transcripts were coded individually by LR in Microsoft Word (MS Corporation, Redmond, WA, USA) and later exported to Microsoft Excel (MS Corporation, Redmond, WA, USA); an initial coding framework was used to guide the coding process following familiarisation with the interview transcripts. Initial interviews were also co-coded by ED to confirm agreement on the coding framework developed; however, this framework was reflexively amended throughout the data collection process. During data analysis, priority was afforded to the ‘voices’ of the participants, which were regarded as the ‘primary source of knowledge’; however, an awareness of our own interpretations was recognised and reflected upon throughout the process. Phase three searched for themes in the data, grouping all relevant data items relating to each potential theme. Preliminary thematic maps were developed to enhance the grouping of data. After phase three, the project team (LR, ED and KH) met to evaluate the potential themes identified. Phase four reviewed the themes, and a refined map of the thematic analysis was produced to analyse how well the themes captured the coded extracts. Phase five further defined and refined these themes, identifying the specifics of each theme and creating sub-themes. The final phase was the complete analysis of the data extracts (quotes, codes and subthemes) identified under each theme.

Data saturation was reached when no new themes, sub-themes or insights were identified from the data and subsequent interviews yielded recurring information that confirmed existing patterns. The initial interviews provided a broad range of ideas related to the study’s research questions, and as data collection progressed, the themes became increasingly rich and consistent. By the 17th interview, thematic consistency had been achieved, with subsequent data only reaffirming the previously identified themes. Thus, data collection was concluded at this point, as the research team (LR, ED and KH) determined that additional interviews would not yield further unique insights relevant to the study’s focus on the experiences of concussion in women’s rugby.

US

American participants were asked to report (yes or no) if they ever suffered a concussion and did not tell anyone. If they selected yes, they were asked to select what reasons caused them to not report: (1) I did not think it was serious; (2) I did not know it was a concussion; (3) I did not want to be pulled from the game/practice; (4) I did not want to be pulled from future games/practices; (5) I did not want to let my teammates down; (6) I would have if it was a less important game/practice; (7) Other. If participants selected ‘Other’, they were provided with a text box to fill in an open-ended response as to why they intentionally did not report their injury. These open-ended responses were thematically analysed using the same coding framework developed above.

## 3. Results

### 3.1. Participant Characteristics

UK and Ireland

A total of 17 participants were purposively sampled after confirming that they met all the sampling criteria. All participants were international rugby union players representing Ireland or the UK countries (England, Scotland and Wales) at the time the data were collected. Participants were also playing in the Premiership (Premier 15s, UK) or representing their province (Ireland) and playing in the AIL (All-Ireland League). The playing positions were separated into forwards (*n* = 11) and backs (*n* = 6). Out of the 17 players interviewed, 16 had experienced at least one concussive injury during their time playing rugby and 10 players had experienced ≥5 concussions (Table 1). Interviews lasted between 29 and 68 min (*M* = 43.17 min, *SD* = 12.1 min) in duration. All interviews were audio-recorded and transcribed verbatim by the lead researcher (LR).

US

From the original cohort of 1037 men and women American Community rugby players, 336 (32.4%) noted that they intentionally did not report a concussion. This sample was further reduced to those who were women who completed the open-ended text box for reason for non-disclosure (*n* = 11, 1.1%). Of these 11 women rugby players, the average age was 29.5 ± 10.6 years (range: 19–56); 100% had a history of diagnosed concussion (range: 1–18).

### 3.2. Thematic Analysis

Four main themes were identified from the research: 1. women rugby players are self-managing SRC; 2. work-related concerns impact on SRC disclosure; 3. players and support staff lack knowledge on SRC management; and 4. poor internal and external communication affect the support players receive when injured. Each of these themes was further divided into subthemes (Table 2).

#### 3.2.1. Theme 1: Women Rugby Players Are Self-Managing SRC

The first theme highlighted that women are often self-managing their own concussive injuries. Subthemes identified within this theme included not recognising concussion as an injury, a lack of support for women who experience a concussion, difficulties in recognising the symptoms of concussion, the time of season (particularly if at a crucial point in the season), the known desire of players to stay playing and players worried about how they will be perceived by others.

Many players did not disclose that they had a concussion to a member of their support/medical team and instead chose to manage their concussion themselves. A range of factors influenced the decision to self-manage a concussion. Some players did not immediately recognise the signs of a concussion when it occurred but retrospectively realised/acknowledged that their behaviours and symptoms were the result of a concussion (Table 2).

Three years ago, there was a certain point in the game I think I had the ball and I fell to the ground and I couldn’t remember falling to the ground. I just remember being on the ground and I was absolutely convinced that I knocked someone’s knee or something like that, … I felt really unwell and I went and asked. I was like, I think I got a concussed on the pitch. I’m pretty sure I was concussed.… I was so confused about why I was feeling the way I was feeling like I had such a bad headache, I felt really sick and they checked the footage and the angles the referee was kind of in the way of all the angles, so they then were like, no, we can’t see anything that suggests you’re concussed.

(P009)

Others stated that they ‘didn’t bother to report my concussion’ as they had previously received a lack of support with the management of a concussion; therefore, they chose to manage their concussion and resulting symptoms themselves without seeking medical support. For some players, the time of season they sustained a concussion influenced whether they would disclose (or try to hide) their concussive symptoms. Players cited ‘big matches’, competing for position and if the end of the season was in sight as influencing their decision. Athletes will seldom want to be removed from the field of play, and all players interviewed highlighted their desire to continue playing if injured. Interestingly, players did discuss being vocal if they noticed that a teammate was displaying concussive symptoms and stated that they would tell a team member to remove themselves or highlight it to the coaching team. Some players perceived that admitting they had sustained a concussion would be a sign of ‘weakness’ or felt that they would be ‘letting the others down’ if they were no longer available to play.

I remember going, I’m just worried I’m letting the team down or I feel, I feel like a wimp saying anything, I feel weak. I did worry as I never wanna look weak. I think especially as a rugby player as you know.

(P004)

#### 3.2.2. Theme 2: Work-Related Concerns Impact on SRC Disclosure

Although the women’s game is changing considerably and many elite women’s players now have some level of contract (and associated payment), it is still quite normal for international players to have another form of employment, and semi-elite players typically work outside of their playing career.

Just the amount of hours that women that play at this level put in…. And working full time and playing rugby at the level it is a lot like I don’t think people realize how much time that we have to put into it and how much energy it takes.

(P014)

Recovery and everything else like that is huge and one of the big ones is definitely sleep. And then the other one I think is actually around food and finding time to eat. The girls that go off and work in the mornings they could be up at 7:00 o’clock in the morning and then not getting home till 10:00 PM after training.

(P009)

The need to work places an additional pressure on some women. Subthemes identified under Theme 2 included financial pressure, risk of losing employment and affecting the ability to work. Participants highlighted that a deterrent to disclosing a concussion was that disclosure may also impact on their ability to work or, depending on the occupation, whether they would be retained in employment.

What I would have noticed is that the major concerns of most individuals who are playing would have been more occupational, (worried) about whether it was going to stop them from working altogether because that was gonna impact them in that way.

(P003)

All of the American athletes included in the study sample were unpaid for their participation in rugby. An additional perceived pressure for these American athletes is that their health insurance is linked to their full-time employment; therefore, if they are unemployed, they are no longer covered by health insurance, creating an added stressor associated with leisure time sport participation.

#### 3.2.3. Theme 3: Players and Support Staff Lack Knowledge on SRC Management

Subthemes under Theme 3 included players unaware of the risk, coaches’ attitudes and the support team lacking awareness or understanding. Concussion education is delivered in rugby, and despite the fact most participants included in this research had undertaken training modules, many of them still had a considerable lack of knowledge of the risks associated with concussion. This may also be linked to the culture in the coaching setup and the emphasis that is placed on providing true education in concussion recognition and management. For example, one player cited that they had to complete online concussion education modules with associated multiple-choice questions at the end of the course and that ‘a few players had the quiz open at the same time and shared answers so we could get it done and dusted quickly (laughs)’ (P008). It was apparent that, despite best practice suggesting a removal from play if signs and symptoms of concussion are recognised, players are still being asked if they ‘want to be removed’ or if they’re ‘ok to stay on the pitch’. Given the known desire of players to keep playing (Theme 1), players should not be asked these types of questions.

There also seemed to be a lack of knowledge or awareness of the signs and symptoms of concussion by some of the medical team supporting players. Some players even requested to come off the pitch because they thought they were concussed (self-disclosure) but were told to stay on by the physiotherapist on the sideline.

She (friend) said to me ‘Well, I can see that you’re concussed’, but I kept going to the physios and the doctors and they kept saying ‘no’, it was like maybe I’m just overreacting. Like I hadn’t been playing rugby that long at that point. It was in my first season of playing for Country and I was like, OK, well, they’ll know better.

(P018)

#### 3.2.4. Theme 4: Poor Internal (Within a Team Setup) and External Communication (Between Teams) Affect the Support Players Receive When Injured

Communication seems to be a key factor in whether a player feels able to disclose a concussion (subthemes: poor internal communication, poor external communication and players not being listed to). Many players gave examples of negative experiences when they had spoken up about concussive symptoms. Some players said they were left to ‘figure it out on my own’ or were dealing with different physiotherapists at different training and match sessions and that an inadequate internal communication system affected continuity of care.

I feel like there just should be more communication between the S&C coaches and the physios, especially like. Sometimes it can be a little bit of a shit show and obviously the S&C is focusing on fit players as well as injured players etcetera, etcetera, yeah, it’s just it’s a lot because with the league, or where the league is at in itself, it is trying to get out as much of an ‘Elite performance’ from us as possible, but where the staff is at the moment, it doesn’t match up to that.

(P014)

Players also highlighted difficulty in external communication between, for example, international camp setups and their clubs, with information on medical care not always shared accurately or frequently (Table 2).

No, it’s not brilliant between them (club and international camp) because there’s a large piece of like who is responsible for you. And then whoever is responsible for you wants to kind of like take over everything, but you’ve got to have some sort of communication between the two.

(P009)

I think it’s that communication between like the international set up and Club, but even between the club staff like. The physios, sometimes I would explain to like, X (coach), ‘I’m in so much pain’, but they hadn’t told her, she didn’t even know (I was injured) and she was selecting me.

(P015)

Players also felt that at times they were not listened to by the medical support teams, which then put them off disclosing subsequent suspected concussions.

I don’t know the communication. I think when it is more full time, hopefully that will improve. But I think it’s such a basic thing that does it is does affect you and it definitely affected me mentally as well because it was just frustrating having this whole thing (injury) and I had to explain it every single time.

(P015)

## 4. Discussion

The primary aim of this paper was to examine the factors that may affect the non-disclosure of SRC in women’s rugby and to gain an understanding of the reasons why players may not disclose their symptoms. Utilising two different methodological approaches across the UK and Ireland and the United States, similar themes were identified. It is clear that the awareness of concussion has improved over the last decade given the greater research focus and media focus that the topic now receives. The majority of players that took part in the research were aware of some of the signs and symptoms of concussion, though some still associated the term with meaning ‘knocked out’ or ‘unconscious’ and had limited understanding of the other signs and symptoms of concussion. Interestingly the players’ declarative management of concussion (following protocols) was very different to how it seemed to be managed in practice. Many players discussed how concussion should be managed but openly admitted to not disclosing symptoms of concussion for a variety of reasons.

A major theme identified in this research was the self-management of SRC by players. There are many concerns with players both ‘self-diagnosing’ and ‘self-managing’ their own concussions. The CISG state that SRC signs and symptoms ‘commonly resolve within days but may be prolonged’ [7]. As such, the graduated RTP they propose suggests a minimum of 24 h per stage leading to a potential RTP within seven days of the initial injury. However, the same consensus statement acknowledges a median RTP of 19.80 days (95% CI = 18.80–20.70 days) [7]. Given the complexity of SRC, the proposed RTP timeline may not allow for full recovery from a SRC. The CISG and individual national rugby union SRC-RTP guidelines clearly require medical clearance before players return to participation in full-contact activities [7]. If players are managing their own concussion, it is unlikely that they are adhering to RTP guidance, and none (in the current study) were seeking medical clearance prior to returning to training. Many players seemed unsure if they had a concussion, highlighting that there is still some need for further education for players and for support staff in recognising and understanding the signs and symptoms of concussion.

In line with previous research (in both men and women), players were concerned with how they would be viewed by fellow players or coaching staff if they ‘admitted’ to having a concussion [44,45]. Players spoke openly about ‘not wanting to let the team down’ or being perceived as ‘weak’ by their own teammates or opposition if they were known to be ‘concussion prone’. Given that it is still possible for players to hide a concussion (unlike a torn hamstring, for example), it is imperative that cultures within sporting organisations and the structures that surround players (teammates, coaches and family/friends) support the disclosure of SRC by creating an environment where players do not experience negative feedback for speaking up.

Though many of the players involved in this research were playing international rugby at the time, the majority of the women included in the current research were in active employment alongside their rugby career. This places not only an additional pressure and less recovery time on the players but also an additional reason not to disclose a suspected SRC. Players spoke of potentially being ‘let go’ from their employment if they knew they had sustained a concussion, and others highlighted that they ‘could not afford to take the time off work’ if their concussive symptoms were known. International women’s rugby is currently transitioning to support more women financially in the game, with many international women’s teams now offering contracts to players; however, in some instances, these contracts are not sufficient to negate the need for additional employment. Coaching staff and support teams involved in rugby should be aware of this additional issue potentially facing women in rugby and ensure that this is considered and discussed in concussion education programmes.

In line with enhancing awareness of concussion, World Rugby has supported the need for greater knowledge of the signs and symptoms of concussion, and all those working in rugby and playing rugby are meant to complete some level of concussion education [46]. It is evident from the current research, however, that some of the players still did not consider the long-term risks of SRC and did not consider a brain injury as ‘serious as breaking a bone’. The support staff surrounding players also have considerable influence on whether a player will disclose a concussion [47]. Research has highlighted that coaches’ attitudes to and knowledge of concussion can significantly impact SRC disclosure by players [48]. To date, the majority of investigations aimed at understanding SRC disclosure have focused on the players themselves [49,50,51,52,53,54,55], their knowledge, attitudes and intention towards reporting. We know that a player’s desire is to play their sport of choice. The onus should not only be on the player to be aware of the signs and symptoms of concussion but also on the teams surrounding them, and the culture should support immediate recognition and removal from play if a concussion is suspected. This research has highlighted that players received negative feedback from some of their coaches when they tried to disclose a concussion and also that, at times, their medical teams did not seem fully aware of the signs and symptoms of concussion.

**Figure 1:**
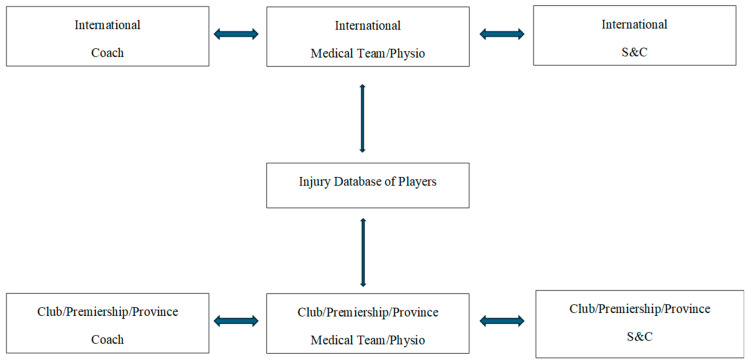
Proposed flow of communication for injury management. Communication needs to flow horizontally (between medical staff and coaching staff) and vertically between players’ clubs and their international camps.

Communication (internal and external) seemed to be a key factor affecting the player’s willingness to disclose a SRC. Enhancing communication and actively listening to players may also increase the likelihood of players discussing their injuries more openly with coaching staff and medical teams [45]. As illustrated in Figure 1, communication needs to flow horizontally (between medical staff and coaching staff) and vertically between players’ clubs and their international camps. At present, there is no centralised system for logging player injury data, and, at times, there is minimal communication between groups (professional silos). This increases the risk of a player ‘playing injured’, as staff are unaware of their existing injuries. One player spoke about the fact that she started a game with ‘the wrong leg strapped’, as no one had written down which leg her injury was on. Previous research from our group found that support teams seemed ill-equipped to manage concussion effectively and lacked an understanding of associated RTP protocols [56,57]. Baugh et al. [58] previously highlighted that the more concussions a player has had, the less likely they may be to disclose a concussion. To enhance injury management, effective communication strategies need to be implemented and the onus removed from players to be the ones responsible for the disclosure of their injury.

### 4.1. Applied Implications

The current research has emphasised the significant role that the entire coaching and support team could play in enhancing disclosure of SRC and the subsequent management of SRC. Although there are clearly still some educational needs, in line with previous research [26,27], a lack of knowledge was not the only factor which may impact a player’s non-disclosure of SRC. Some of the players included in the study were very aware that they were potentially suffering from concussive symptoms yet actively chose not to disclose their symptoms for a variety of reasons, including their desire to play, fear of how they would be perceived or fear of how a concussion diagnosis may impact on their employment status. The applied implications of this study are that it may inform coaching and support staff in women’s rugby that there are a number of factors that may affect a player’s disclosure of a concussion and ensure that strategies are put in place to create a culture and environment where a player feels supported in disclosing a potential concussion. This study also highlights the complexity of the management of concussion and the need for objective measures to diagnose concussion to prevent players potentially hiding their symptoms. Coaches and support teams are still asking the players themselves if they are ‘able to carry on’ after a suspected concussion rather than consistently employing the ‘if in doubt, sit them out’ approach. Understanding the factors that influence a player’s disclosure of a SRC will assist in creating protocols and environments that enhance and support player welfare.

### 4.2. Limitations and Future Directions

As highlighted previously, women’s rugby is in transition and changing rapidly due to its increasing global popularity. Therefore, the themes identified through this body of research are representative of a particular period in time. A potential limitation of the present study was that it used two different approaches (one-on-one interviews in the UK and Ireland and open-ended survey questions in the US), which meant the level of detail varied considerably between the two approaches. This qualitative investigation has highlighted a commonality in the reasons for SRC non-disclosure across women’s rugby in the UK and Ireland and the United States. Future research should seek to understand coaches’ and support teams’ knowledge and understanding of concussion management in women’s rugby. Additionally, considerably more quantitative and qualitative research in women’s rugby is required to enable us to gain an understanding of the differences in concussion prevalence, symptomology and recovery between men and women.

### 4.3. Conclusions

This research has highlighted several factors that may impact women rugby players’ disclosure of SRC regardless of country of origin (the UK, Ireland or the US) and access to concussion care. Coaches and management teams should be aware of these reasons, which may enhance how they discuss and manage concussion. There is a clear need for further education, particularly for players and support teams involved in women’s rugby, on concussion signs, symptoms and management and for strategies to create environments where women can openly discuss their concussion concerns and improve effective identification and reporting of SRC.

## Figures and Tables

**Table 1:** Participant characteristics.

Participants	UK and Ireland	United States
*n*	17	11
Age (years)	29.6 ± 5.6	29.5 ± 10.6
No. of concussions	4.5 ± 2.8	5.4 ± 5.4

**Table 2:** Developed themes, subthemes and example quotes: factors affecting SRC non-disclosure in women’s rugby.

Theme	Subtheme	Example Quote
1. Women rugby players are self-managing SRC	Not recognising concussion as an injury	And so, it wasn’t a knock-out but I definitely wasn’t OK. I remember I was crying on the bench running around because I didn’t know which way I was running, and I was just completely gone… I believe my [international] career wouldn’t have been as long if what was known about concussion was known then. (P004)
	Lack of support	So, you’re very much left. I was very much left on my own at that stage [after concussion]. And then just kind of I suppose use my own initiative… even when I’ve had surgery, I had to go to the lecturers in the college, there was a sport rehabilitation going on, so I went to them and said look, I have had this surgery I’m not really sure what I’m supposed to be doing. (P001)
	Recognising symptoms	I think that that’s a key part of educating players now, it isn’t necessarily their own symptoms and their own recognition it’s actually, recognizing someone else being concussed (P005)
	Time of season	The season was over so I knew I would have multiple months before rugby started back up again. I took a 2-month workout break after suffering the concussion. (P018)
	Desire to play	Oh and I guess it’s the part of you as a player, that really wants to play at the weekend and if it’s, I think especially if it’s a big game or a, you know, an important game or one that’s really competitive and you want to be involved in or if you’re trying to make a team or if you want to be performing. And then I think that there is a part of an athlete that might say you know like you really can’t miss this because it might affect an opportunity elsewhere or you know. (P001)
	Worried about perception of others	Never let anyone know—don’t show weakness. (P021)
2. Work-related concerns impact on SRC disclosure	Financial pressures	I couldn’t afford not to work…. (P020)
	Risk of losing employment	The major concerns of individuals who are playing would have been more occupational.. and whether it was going to stop them from working together was gonna impact them in that way. (P003)
	Affecting ability to work	[after concussion]… I couldn’t handle it and I said to one of my bosses and I I told them, they said go home and I think I was off work probably for about a month maybe or so maybe a bit longer but I even remember when I was trying to drive later on… You know just even that kind of foggy and you know so it was just it was just kinda housebound really. (P006)
3. Players and support staff lack knowledge on SRC management	Players unaware of risk	I don’t think I knew enough about what concussion was then to know that I was [concussed]. I knew that I was in pain ‘cause I broke my nose. Uhm, so you know, did I want to be removed? No, because I’m competitive and I wanted you know, and we get knocks and bangs and bumps and bruises in rugby. Was I misleading the medics when they asked me did I feel OK? I think it’s hard to know whether you are OK (P005)
	Coaches’ attitudes	Coach said to ‘suck it up’ (P026)
	Support team lacking awareness/understanding	Yeah, it wasn’t even bad. Like, it’s kind of complicated because just before the Six Nations, I got concussed for the first time and I was kind of ill throughout the whole period and I had headaches, but they [medical team] put it down to being ill. (P015)
4. Poor internal and external communication affect the support players receive when injured	Poor internal communication	And so, I, to be honest, I had a very bad experience with that situation in general, how it was looked after, the follow up who I was even supposed to be in contact with and how the physio sessions were even going to be managed so I end up being quite stressed about the whole thing. (P008)
	Poor external communication	It was really poor even communication wise. Now I must say my [international] Coach at the time rang me straight away and was like ‘we’ll get this all [support] in place’. But then when it came to the staff at club level… that didn’t happen. (P008)
	Players not being listened to	It kind of depended who you are and how much you knew maybe about your own body or about your own previous injuries or how to manage training or how to manage would also depend whether the physio would almost listen to your self-prescription. (P010)

## Data Availability

Supporting data are available within this paper.
